# The Relationship Between Race and Ethnicity in Post-Surgical Discharge Disposition

**DOI:** 10.1093/geroni/igaa057.459

**Published:** 2020-12-16

**Authors:** Caitlyn McFadden, Coco Victoria Tirambulo, David Lieberman, Mindy Fain

**Affiliations:** 1 The University of Arizona Center on Aging, Tucson, Arizona, United States; 2 University of Arizona College of Medicine - Geriatrics, General Internal Medicine and Palliative Medicine, Tucson, Arizona, United States; 3 University of Arizona Center on Aging, Tucson, Arizona, United States

## Abstract

Racial and ethnic disparities have been reported regarding outcomes of intermediate or high-risk surgical (IHRS) procedures. This study aimed to assess whether or not these disparities exist with respect to post-procedural discharge disposition (DD). EMR chart reviews of patients (≥65 years old) undergoing IHRS were conducted, 2016-2019 in Tucson, AZ. Race and ethnicity were reported as American Indian/Alaskan Native; Black or African American; More-Than-One-Race; Native Hawaiian/Pacific Islander; White/Caucasian; Unknown/Not Reported, Hispanic/Latino; Non-Hispanic/Latino; or Unknown/Not Reported. DD was reported as to home, home with home health, rehabilitation center, skilled nursing facility (SNF), death, or other. Kruskal-Wallis tests were performed to determine the association with DD. Of the 161 patients (mean age: 74.7±6.9, [60-91], 53.4% male) assessed, 15.4% were discharged to a facility other than home or home with home health. Ethnicity was significantly associated with discharge disposition (p<0.005). Non-Hispanic/Latinos (66.1%) were more likely to be discharged home compared Hispanic/Latinos (14.8%), while those Unknown/Not Reported were more likely to be discharged to a SNF. Although race was not significantly associated with DD (p<0.062) due to sample size, it is clinically important to consider as 59.9% of White/Caucasians were more likely to be discharged home compared to 1.6% of American Indian/Alaskan Natives; 1.6% Black or African Americans; 5.7 of More-Than-One-Races; and 12.0% Unknown/Not Reported. We demonstrated ethnic disparities affect postoperative DD. Although racial disparities were not significantly associated with DD, anticipating the impact of race and ethnicity to facilitate discharge planning that meets the goals of the older patient is crucial to care.

